# Case Report: Diagnosis and Assessment of Cure Approaches for Acute Schistosomiasis in Pre-School Children

**DOI:** 10.3389/fimmu.2021.624736

**Published:** 2021-05-12

**Authors:** Marta G. Cavalcanti, Délia Celser Engel, Aline Fernandes de Araujo Cunha, José Mauro Peralta

**Affiliations:** ^1^ Serviço de Doenças Infecciosas e Parasitárias, Hospital Universitário Clementino Fraga Filho, Universidade Federal do Rio de Janeiro, Rio de Janeiro, Brazil; ^2^ Departmento de Imunologia, Instituto de Microbiologia Paulo de Góes, Universidade Federal do Rio de Janeiro, Rio de Janeiro, Brazil

**Keywords:** acute schistosomiasis, praziquantel, real-time PCR, POC-CCA, young children

## Abstract

Acute schistosomiasis (AS) manifests with a broad spectrum of clinical features in pediatric populations. Diagnosis may be difficult in the absence of detectable numbers of eggs. As a result, new approaches may be required to achieve an accurate diagnosis. Optimal praziquantel (PZQ) treatment regimen for young children is debatable. Also, the post-treatment response is still poorly evaluated due to the lack of reliable markers. A group of 6 children (a toddler and 5 pre-school children) and one pre-adolescent were investigated for AS clinical manifestations and followed-up for two years after treatment. Ova detection was performed by Kato-Katz (KK) and presence of *Schistosoma mansoni* DNA was assessed by real-time PCR (rt-PCR) in stool samples. IgG and IgE anti-*Schistosoma* levels and urinary antigen were detected by ELISA and point-of-care circulating cathodic antigen (POC-CCA) testing in serum and urine, respectively. AS clinical symptoms were present in 5/7 (71.4%) of the infected children, and hypereosinophilia was detected in all of them. Ova detection and serology were positive in only 3/7 (44.9%) and 4/7 (57.1%), respectively. However, real-time PCR (rt-PCR) showed the presence of *Schistosoma* DNA in 6/7 (85.7%) of the cases, and urinary antigen was detected in all infected children. The long-term follow-up after treatment with three doses of PZQ (80mg/kg/dose), showed high cure rates (CR) as demonstrated by the DNA-based assay as well as reduced levels of side effects. CR based on urinary antigen detection ranged from 28.6 to 100%, being the highest CR due to double testing the 2-year post-treatment samples. The results suggest that high dose and repeated treatment with PZQ might be effective for AS in young children. Also, new laboratory markers should be considered to diagnosis and monitor the drug response.

## Highlights

The study investigates AS in a group of young children exposed to *Schistosoma* in a low endemic area. Clinical and laboratory markers were used for long-term follow-up to assess the response to a regimen of high dose and repeated intakes of PZQ.

## Introduction

Schistosomiasis is a water-borne disease that has a substantial public health impact in low income countries. *Schistosoma* infections present as acute and chronic forms. Acute schistosomiasis (AS) often occurs in non-immune individuals living in non-transmission areas, such as travelers (1–3). Outbreaks among travelers may have high infection rates, and AS signs and symptoms are reported in 73 to 100% of cercaria-exposed individuals (1, 4). Although up to 40% of infected individuals are asymptomatic, specific symptoms are found in 24 to 66.4% which may subside without treatment. Clinical outcomes are usually benign, but severe and atypical disease may occur (4–7). Eosinophilia is reported in up to 82% of the cases, and is considered an early laboratorial marker of the disease (3, 5, 8). *S. mansoni* infection may be classified as light to heavy based on the parasite burden or egg counts (9). Heavy infections are usually associated with increased clinical severity due to host allergic responses, although high morbidity can also be seen in light/mild infections. Therefore, laboratory-based diagnosis is fundamental for accurate identification of AS cases (2, 4, 10–14).

AS involves two distinct phases related to *S. mansoni* biological cycle. During early infection (prepatent phase: 3-7 weeks post exposure), immature forms migrate from the sites of skin penetration to several host systemic compartments, such as the lung, liver, gut, and visceral peritoneum until harboring mature bi-gender forms in the mesenteric veins (15). In this early phase, eggs are not detectable until ≅40 days post-exposure and alternative diagnostic approaches are usually applied other than parasitological tests (14, 16). Routinely, indirect diagnostic assays such as serology and other laboratory markers like eosinophil counts are mostly used (1, 2, 4, 17, 18). However, both diagnostic approaches have poor sensitivity during the early phase of infection (19). Seroconversion may be absent until 6 weeks post-exposure, depending on the antigen used. Also, serum reactivity may occur late in an acute infection (15, 20, 21). Eosinophil counting is another unreliable marker since eosinophilia may not occur, and its absence does not rule out acute infection (20). Occurrence of eosinophilia ranges from 73 to 100% among acutely infected individuals (4, 18). In the late acute phase (> 45 to 60 days post exposure), oviposition occurs, and eggs can be detected by parasitological methods. However, mild intensity infections with low parasite loads may not be detectable (12, 13, 15, 22). Studies in immigrant populations have shown that egg positivity is low among children living in non-endemic areas (23). Not only diagnosis is challenging, but the response to PZQ has also been poorly assessed by using reference methods and/or laboratory markers. For instance, serum reactivity may remain positive for long periods even after effective anti-helminthic therapy, leading to difficulties in distinguishing active and cleared infections (23–25). Alternative diagnostic tools, such as tests to detect *Schistosoma* antigens and DNA, are promising approaches for the diagnosis of schistosomiasis (2, 10, 13, 16). Studies on travelers and immigrant populations have shown that the detection of *Schistosoma* antigens and DNA has the potential to improve AS diagnosis, treatment response and determination of cure rates (2, 14, 24).

Although considered a safe and efficacious drug, PZQ has some important limitations when used for AS treatment. For instance, young immature parasite forms are usually resistant to PZQ, and clinical manifestations become exacerbated in about 50% of treated individuals (26–28). Also, since chronicity is not preventable, delayed PZQ administration is recommended by some investigators (17, 18, 20). Drug posology and dosage are still debatable since discrepant results in chronically infected under-aged children have been reported (29, 30). In fact, the ideal drug regimen to treat AS is still a challenge in any age group, which may contribute to treatment failure and progression to chronic disease (18). Scarce clinical information on PZQ pharmacokinetic-pharmacodynamics and the lack of a proper formulation suitable for young children may also contribute to drug response failure in this group of patients (29). Early treatment in chronically infected children living in endemic areas seems to be essential to decrease morbidity and the risk of chronic sequelae. However, early treatment of young children living in non-endemic areas is another not well-addressed issue (31). Hence, AS treatment needs urgent revision and improvement.

Here, we report the occurrence of AS in seven children exposed to *Schistosoma*-contaminated water in a low endemic area (LEA). Clinical and laboratory approaches were used to investigate their potential to base accurate diagnosis and to evaluate response to high dosage PZQ therapy.

## Material and Methods

### Ethical Statement

The population investigated was informed of the protocol and written informed consent was obtained from children legal guardians. The study was approved by the Hospital Clementino Fraga Filho, Universidade Federal do Rio de Janeiro (HUCFF/UFRJ) Ethics Committee (n°058/09).

### Patients

From November to December 2015, six pre-school children and a teenager from the same family received a diagnosis of AS. In September, they (four boys and three girls) were exposed to cercaria-contaminated water in cement tanks that collected water from natural springs in Sumidouro city, Rio de Janeiro, Brazil. All of the children lived in a non-endemic area of Rio de Janeiro (Nova Friburgo city, RJ, Brazil). Fecal, blood and urine samples were obtained from all participants prior to and at three different periods after chemotherapy.

### Parasitological and Immunological Diagnoses

*S. mansoni* infection was determined based on Kato-Katz tests on three independent fecal samples/individual (2 slides/sample). Specific IgG and IgE anti-adult worm membrane soluble antigen (SMMA) were measured in serum by using an ELISA (SMMA-ELISA) as described previously (6). The results were expressed in arbitrary units (A.U.) that were calculated by dividing the sample OD by the daily cut-off OD value. Values > 1.0 were considered positive.

### Real-Time PCR for *Schistosoma* DNA Detection

Real-time PCR was used for DNA detection in fecal samples. Probes and primers targeting the cytochrome c oxidase subunit 1 (COX) gene in the mitochondrial genome of *S. mansoni* were designed as previously described (14). DNA was extracted from fecal samples by using the Fast Prep DNA kit (MP Biomedicals, CA, USA) according to the manufacturer’s instructions and purified using the QIAquick PCR purification kit (QIAGEN, Hilden, Germany). The reaction was composed by prepared using 5 μL of eluted DNA as a template, mixed with 2.0 pmoles/μL of each *S. mansoni* primer (SMCYT748F and SMCY847R), 2.5 pmoles/μL of SMCY785T detection probe, 11.0 μL of Platinum qPCR SuperMix Rox-UDG (Invitrogen) and 2.3 μL of deionized water to make a final volume of 20 μL. The amplification conditions were 15 min at 95°C followed by 40 cycles of 15 s at 95°C and 1 min at 60°C. Amplification, detection, and data analyzes were performed using the 7500 Real-Time PCR System (Applied Biosystems, CA, USA). DNA amplification was considered positive when Ct values were < 38.

### Point-of-Care Circulating Cathodic Antigen (POC-CCA) Test

Urine samples obtained pre and post-praziquantel treatment were tested by a rapid test. POC-CCA test1 (batch 50173) were performed in all pre and post-PZQ treatment samples, according to the manufacturer’s instructions (Rapid Medical Diagnostics, Pretoria, South Africa). A second batch of POC-CCA test (POC-CCA test 2; batch 17522062), upgraded by the manufacturer in 2017 was also used, and conducted only on the last post-PZQ treatment urine sample obtained from each patient. Results were scored as: “0” if the result was negative, and positive tests were graded as “1+” or trace, “2+” and “3+” as described by Coelho et al. (16).

### Schistosomiasis Diagnosis Criteria

Diagnosis of schistosomiasis was based on a positive result for egg, antibody, DNA and/or urinary antigen detection.

### Praziquantel Treatment and Cure Assessment

Patients received orally, in 2 divided doses, 80 mg/kg/day of praziquantel (PZQ; Farmanguinhos, FIOCRUZ, Rio de Janeiro, Brazil). Children aged ≤ 5 years received PZQ tablets that were crushed and mixed with fruit juice and/or sweet snack of the child preference (32). The same treatment was repeated after 30 and 45 days, respectively, after the 1^st^ dose. PZQ was administered by the parent/guardian under supervision of the assistant physician. Acceptability of the tablet was assessed directly during and up to one hour after PZQ administration. Parents/guardians were instructed to report eventual cases of intolerance (nausea, choking or vomiting within 24 hrs post-drug administration). The response to PZQ was evaluated after 4, 13 and 22 months post-treatment.

## Results

Subject ages varied from 1.7 to 14 years old (mean: 5.8 ± 4.1). Symptoms onset and/or laboratory confirmation (asymptomatic case) ranged from 53 to 78 (mean ± std: 65.3 ± 9.4) days post-exposure (dpe; **Table 1**). Two five-year-old boys (cases 1 and 2) were the first to manifest moderate to severe symptoms. Case 1 developed vomiting and liquid diarrhea at 35dpe, followed by daily fever (38-39°C) on day 36. He also had a history of abdominal pain, occasional non-productive cough and wheezing that occurred from 48 to 54dpe in addition to liquid diarrhea accompanied by mucous and blood. Parents mentioned that generalized pruritus and erythematous rash on the torso were observed after water exposure. Leukocytosis (mean: 22.600 ± 5.188/mm^3^), eosinophilia (total: mean: 5.994 ± 4.197/mm^3^; relative, mean: 26.3 ± 19.6%) and thrombocytosis were observed 45 to 53 dpe. Both eggs and DNA were detectable in fecal samples at 53 dpe in addition to IgG anti-SMMA and *Schistosoma* cathodic antigens in serum and urine, respectively (**Table 1**). Case 2 had intense pruritus immediately after water exposure but no exanthema. At 19-20 dpe, intermittent fever (37.8-38.9°C) initially occurred for 9 days and liquid mucous diarrhea was observed in the following week (**Table 1**). Then, after a week without symptoms, the child had fever (39.5°C) again for another 8 days while being treated with amoxicillin, followed by another episode on 49 dpe. Leukocytosis, eosinophilia and thrombocytosis were detected on 52 dpe. Fecal samples showed eggs and DNA on 53 and 61 dpe, and urinary antigen on 53 dpe, without any detectable levels of IgG or IgE anti-SMMA (**Table 1**). Case 3, a four-year-old boy also presented with a febrile syndrome, and progressed with various episodes of vomiting and diarrhea, decreased appetite and dehydration, followed by non-productive cough. Because of his general condition and the severity of symptoms, he was hospitalized for 48 hours. Cases 4, 5 and 6 were girls under five years old who had mildly symptomatic disease, presenting with low fever, abdominal pain with/without vomiting and diarrhea (**Table 1**). One single case (Case 7) presented with no symptoms. Leukocytosis and eosinophilia were the major laboratory findings in mildly symptomatic children, and anemia and thrombocytosis were diagnosed in one child in this group. None of the mildly symptomatic/asymptomatic children (4/7) showed egg excretion different from moderate/severe cases. *Schistosoma* DNA was detectable in 6/7 children and all seven children presented CCA in urine (**Table 1**). During the period of 53 to 86 dpe, IgG anti SMMA reactivity was shown in 3 children presenting asymptomatic or mild/moderate disease. All four IgG negative children were mildly symptomatic (**Table 1**).

**Table 1:** Baseline clinical characteristics of acute schistosomiasis cases in children, time of infection and results of laboratory testing.

Case ID	Age in years	Clinical Presentation	Days Post-Infection	KK	ELISA IgG (AU)	Real-Time PCR (Ct)	POC-CCA
**AS01**	5	Pruritus, cercarial dermatitis; fever; vomit, abdominal pain, bloody diarrhea and cough. Leukocytosis. Hypereosinophilia	53	Pos	1.0	23.08	2
**AS02**	5	Pruritus, fever; liquid diarrhea. Leukocytosis Hypereosinophilia	53 61	Pos -	0.8 -	NR 27.35	2 -
**AS03**	4	Abdominal pain, diarrhea, vomit, fever. Non-productive cough. Leukocytosis. Hypereosinophilia. Hospitalization.	61	Pos	0.7	23.79	3
**AS04**	1.7	Fever, Abdominal pain and diarrhea. Leukocytosis. Hypereosinophilia	61 71 86	Neg Neg Neg	0.8 0.3 -	NR 37 NR	2 3 2
**AS05**	3	Fever, mild diarrhea; Hypereosinophilia	78	Neg	0.4	34.45	1
**AS06**	3	Fever, diarrhea, vomit and abdominal pain. Leukocytosis. Hypereosinophilia	78 86	Neg -	1/40* 2.1	NR NR	2
**AS07**	14	Asymptomatic	61 71	- Neg	1.2 -	NR 33.05	1 -

-, not done; NR, Not Reactive; Parasitological Method, Kato-Katz. Pos, positive; Neg, negative; A.U., Arbitrary Units; ELISA IgG and IgE Positive ≥ 1 AU. Negative < 1 AU; Ct, Cycle Threshold (real-time PCR); Ct < 38, Reactive; Ct >38, Not Reactive; POC-CCA: 0, negative; 1, positive very weak (trace); 2, positive weak; 3, positive strong; Hypereosinophilia: > 500/mm^3^.

All children complied with the treatment, although 2/7 manifested transient side effects like nausea and vomiting. Later, both patients received a supplementary single oral dose of PZQ (80 mg/kg/day) without manifesting any symptoms. Ceasing of clinical symptoms and absence of egg excretion were observed during the 2-year-follow-up. *Schistosoma* DNA remained undetectable in all but one child during the entire follow-up period, indicating a cure rate of 85.7% (6/7) (**Table 2** and **Supplementary Figure 1**). This child had no egg excretion but remained reactive according to rt-PCR at 22 months post-therapy. There was no history of re-exposure. A single dose of PZQ (80 mg/kg) was indicated for re-treatment, but the child presented drug intolerance to a single oral dose (80 mg/kg/day) and missed the follow-up. Urinary antigen was not detectable in 3/7 children during the first four months of the post-treatment period. However, POC-CCA test remained positive in 4/7 children, being very weakly positive in 3/4 children (trace; **Table 2** and **Supplementary Figure 1**). POC-CCA test became negative in 2/7 (28.6%) at 22 months post-PZQ therapy. Nonetheless, persistent POC-CCA reactivity was found with variable intensities in sequential samples in 3/7 and 5/7 children at 13 and 22 months post-treatment, respectively. In addition, when tested with a second batch of POC-CCA, no reactivity was detected in urine samples collected at 22 months post-PZQ, including previously reactive samples (**Table 2** and **Supplementary Figure 1**). Also, 5/7 children presented a 3.9-fold increase in IgG levels when compared to pre-therapy baseline levels in the short-term follow-up. Most of the children maintained serum reactivity up to 22 months post-treatment (**Table 2** and **Supplementary Figure 1**). At four months post-therapy, only one individual (1/7) showed increased IgE levels. Late seroconversion was detected in three children at 13 months after drug use (**Table 2**).

**Table 2:** Diagnosis pre-treatment and response to treatment follow-up.

Diagnostic Method	AS01	AS02	AS03	AS04	AS05	AS06	AS07
**Parasitological** **Pre-treatment**	**Pos**	**Pos**	**Pos**	Neg	Neg	Neg	Neg
**Post-treatment** **4 months**	Neg	Neg	Neg	Neg	Neg	Neg	Neg
**13 months**	Neg	Neg	Neg	Neg	Neg	Neg	Neg
**22 months**	Neg	Neg	Neg	Neg	Neg	Neg	Neg
**ELISA**							
**Pre-treatment: IgG/IgE**	**1.0**/0.2	0.8/0.1	0.7/0.1	0.8/0.1	0.4/0.6	**2.1**/0.1	**1.2**/0.1
**Post-treatment: IgG/IgE** **4 months**	**2.7**/0.9	**3.5/2.2**	**2.0**/0.7	**4.1**/0.1	**1.8**/0.7	0.6/0.1	0.3/0.7
**13 months**	**2.0/1.1**	**2.5/1.7**	**1.8**/0.8	**2.0/1.1**	**1.1/1.7**	0.4/0.6	0.02/0.70
**22 months**	**3.0**/0.4	**2.9/1.3**	**1.8**/0.8	**2.3**/0.4	**1.1/.1.3**	0.09/0.1	0.1//1.3
**Real-time PCR (Ct)**							
**Pre-treatment**	**23.08**	**27.35**	**23.79**	**37**	**34.45**	NR	**33.05**
**Post-treatment** **4 months**	NR	NR	NR	NR	NR	NR	NR
**13 months**	NR	NR	NR	NR	NR	NR	NR
**22 months**	NR	NR	NR	**33.29**	NR	NR	NR
**POC-CCA**							
**Pre-treatment**	2	2	3	3	1	2	1
**Post-treatment** **4 months**	1	0	2	0	1	1	0
**13 months**	0	0	2	2	1	0	0
22 months*	1	0	1	1	1	1	0
22 months**	0	0	0	0	0	0	0

NR, Not Reactive; A.U., Parasitological Method, Kato-Katz. Pos, positive; Neg, negative; Arbitrary Units; ELISA IgG and IgE Positive ≥ 1 AU. Negative < 1 AU; Ct, Cycle Threshold (real-time PCR); Ct < 38, Reactive; Ct >38, Not Reactive; POC-CCA: 0, negative; 1, positive very weak (trace); 2, positive weak; 3, positive strong; * represents POC-CCA test1 (batch 50173); **represents POC-CCA test 2 (batch 17522062). The positive results in each test are in bold.

## Discussion

The numbers of newly diagnosed schistosomiasis cases are increasing in non-endemic areas due to tourism and to immigration from transmission areas (2, 11). Nonetheless, clinical and laboratory diagnosis of AS caused by any *Schistosoma* species remains challenging for every age group, including the pediatric population (16, 19, 33, 34). Usually, clinical presentations of AS, along with severity and long-term disability in adult travelers are described in detail. In contrast, information on the characteristics of the disease in young children is scarce. Data show that 54-100% of infected adult travelers may present clinical manifestations of acute disease (1, 4). Amongst the most frequent manifestations, fever (68-100%), abdominal pain (96%), diarrhea (90%) and cough (78-86%) predominate, with small variations depending on the *Schistosoma* species involved (4, 7, 17, 18). In the present study, more than 70% of the children (ages: <2-5 years old) presented moderate to severe manifestations of AS over a period of 6-8 weeks post-exposure to *S. mansoni* cercaria acquired in a LEA. Acute asymptomatic infection was also detected in two children, one being a pre-school child. Although direct measurement of *Schistosoma* infection of the intermediate hosts failed to demonstrate active infection (personal communication), the area has been well-known for its low endemicity since 2000 with presumably low transmission rates (35). Despite that, most children exposed to low parasite burdens develop a broad range of manifestations, including severe disease. Fever, abdominal pain and diarrhea are the major symptoms reported. However, the presence of blood in stool indicates increased severity of disease amongst pre-school children. Although the clinical evolution required hospitalization in one case, fading of symptoms and lack of sequelae and/or chronicity occurred after a follow-up of over two years after treatment. These data suggest that clinical manifestations and outcomes might be independent of the parasite load.

Mostly, acute infection diagnosis relies on clinical/epidemiological data, parasitological tests and/or serology. However, since these methods present limitations, AS may be misdiagnosed (12, 13, 22). Unrecognized AS should not be underestimated, since it may result in high severity and chronicity with increased disability (5, 6, 36). Studies on pre-school children (<5 years of age) living in endemic areas show that early infection is associated with an increased risk of disease progression within three months of exposure (37). As a result, the assessment of active infection with more sensitive diagnostic approaches would be of paramount importance to allow early treatment and to prevent disease progression. In the present case series, time post-exposure exceeded 7 weeks, correlating with the oviposition phase. However, only moderate/severe cases had detectable egg excretion. Asymptomatic/mildly symptomatic subjects had no detectable eggs, probably as a result of low parasite load, undetectable by parasitological methods. Moreover, serology demonstrated reactivity to adult worms in almost half of the children investigated. On the other hand, molecular methods identified early acute infection induced by both mature and immature forms of *Schistosoma* in all or most of the cases. Our results demonstrate that both molecular tests, i.e. POC-CCA and the DNA assay, allowed diagnosis of early infection (<2 months post-exposure). Also, both urinary antigen and DNA detection overcome the positivity of the parasitological method and serology. The present data indicates that early detection of schistosomiasis by molecular assays may improve AS diagnosis in young children.

There is an increased risk of long-term complications in the pediatric, adolescent and adult population who have travelled to or lived in endemic areas exposed to *Schistosoma*-contaminated water, if left untreated. Nonetheless, these individuals usually respond well to drug therapy, including pre-school-aged children (6, 29, 38). Hence, early diagnosis, aggressive treatment and follow-up care should be the key management approaches for schistosomiasis (6). PZQ is the only choice for the prevention and treatment of schistosomiasis. The standard dose of 40 mg/kg body weight is used in a single application for school and adults at the community level through mass drug administration, as well as a therapy to reverse acute and early chronic schistosomiasis in travelers and migrant groups. However, PZQ only achieves 70 to 80% of drug effectiveness in school-children and adults; this is even lower in pre-school-aged children. Inadequate dose regimen, poor adherence due to the non-palatable characteristic of the drug, and scarce knowledge of the pharmacokinetics and pharmacodynamics of PZQ in young children may be important issues related to drug failure in this group (39). Recently, compelling data suggest that increasing the traditional dose may be beneficial in the chronically infected pre-school-aged children population living in transmission areas (29). Also, a new formulation such as orally dispersible tablets (ODT) of levo PZQ had its bioavailability determined in a phase I study. But results showed that there is still a need to determine an adequate dose for the pediatric group, considering that data on effective regimens and new drugs in acutely infected children remain limited (39). Herein, our results suggest that an anti-helminthic drug regimen including a high dose of PZQ (80 mg/kg) and more than a single oral dose might be efficient to treat acute schistosomiasis in the pediatric and adolescent group. Large clinical studies addressing effective drug posology during pre and post-patent AS are not available, and the lack of long post-therapy follow-up assays has compromised the establishment of recommendations for the management of the disease in all age groups, particularly in children (34). Nonetheless, further studies on new drug formulations and regimens are still necessary.

Assessment of the drug response in AS is usually based on traditional diagnostic approaches. In the group of egg excretors, no detection of eggs in a stool sample can occur very early post-treatment. However, in persistent infection or in cases of reinfection, the absence of egg detection does not rule out active schistosomiasis. Eventually, this will compromise decisions like the need for re-treatment. In the series investigated here, the evaluation of the therapy response based on KK showed no egg excretion in all three egg-positive individuals. Samples remained negative until 22 months, suggesting excellent drug efficacy. However, *Schistosoma* DNA was present in a fecal sample from an individual without egg excretion in the long-term follow-up. DNA late positivity in egg-negative individuals may occur post-PZQ administration as shown previously by our group (40). Detection of DNA persistence suggests an ongoing active infection, resulting from continuous DNA release from tissue-trapped eggs or single gender (female)-induced infection. Either way, DNA-reactive individuals that fail to respond to a single dose of therapy can achieve parasitological cure after re-treatment (40). Conversely, the assessment of the drug response by POC-CCA testing showed higher numbers of false positive results. Urinary antigen remained detectable in most of the treated children up until two years post-PZQ administration.

Assessment of *Schistosoma* infection diagnosis by urinary antigen detection proved to be less suitable to be used in low and non-endemic areas because of the low accuracy of POC-CCA testing. Antigen detection assays need standardization, evaluation of performance in community and institutional settings and of reproducibility between different batches (14, 41–43). In the present study, the findings showed false positive results and fluctuation of band intensity scores and discordant results for the same sample tested by using different POC-CCA batches. The data suggest that POC-CCA testing may not be reliable to be used as a marker for evaluation of drug response, in agreement with findings of other investigators (41–44).

Altogether, AS presents with a broad spectrum of manifestations with high morbidity and a potential risk of chronicity. In cases with no egg detection and the absence of seroconversion, alternative diagnostic approaches become essential. The present study demonstrates that molecular diagnosis improved the detection of AS in a group of young children. The findings also suggest that PZQ posology adjusted to a higher dose and more than one intake was effective in pre-school children, resulting in parasite-free status in a period of two years post-treatment, in most cases. After drug use, egg-negativity in the short-term and persistent serum reactivity in the long-term follow-up of schistosomiasis may hide treatment failure. Therefore, the use of more reliable markers of the drug response, such as the DNA assay, may result in improvements in the assessment of cure.

## Data Availability Statement

The original contributions presented in the study are included in the article/**Supplementary Material**. Further inquiries can be directed to the corresponding authors.

## Ethics Statement

The studies involving human participants were reviewed and approved by Hospital Clementino Fraga Filho, Universidade Federal do Rio de Janeiro (HUCFF/UFRJ) Ethics Committee (n°058/09). Written informed consent to participate in this study was provided by the participants’ legal guardian/next of kin.

## Author Contributions

MC and DE gave medical assistance to the study population and collected the data. AC and MC performed laboratory diagnosis. JP and MC analyzed the data. MC wrote the draft of the manuscript, and JP reviewed all versions of the manuscript and helped to shape it up. All authors contributed to the article and approved the submitted version.

## Funding

This work was supported by the Brazilian National Council for Scientific and Technological Development (Grant CNPq/PROEP 400107/2011-2) and Fundação de Amparo a Pesquisa Carlos Chagas Filho, Rio de Janeiro, Brazil.

## Conflict of Interest

The authors declare that the research was conducted in the absence of any commercial or financial relationships that could be construed as a potential conflict of interest.

## Supplementary Material

Supplementary Figure 1 Schematic representation of *Schistosoma* DNA, IgG and urinary antigen detection pre and post-treatment response during short and long-term follow up. Fecal samples from children treated with three sequential doses of PZQ (day 0, 30 and 45) were tested by real-time PCR **(A)**. Serum **(B)** and urine **(C)** samples were analyzed by ELISA-IgG anti-SMMA and POC-CCA, respectively. Ct, Cycle Threshold (real-time PCR); Ct < 38, Reactive; Ct >38, Not Reactive; A.U., Arbitrary Units; ELISA IgG, Positive ≥ 1 AU. Negative < 1 AU; POC-CCA, 0, negative; 1, positive very weak (trace); 2, positive weak; 3, positive strong. Arrows indicate individuals with persistent reactivity post-drug use during long-term follow-up. In the 1^st^ row, dashed lines represent the cut-off cycle threshold value. In the 2^nd^ row, ELISA’s values > 1.0 were considered positive (dashed line). In the 3^rd^ row, dashed lines correspond to results of the last urine sample (collected after 22 months of treatment) tested by POC-CCA test 2 (see *Material and Methods*).
