# Supplementary figures and images for: Case Report: Diagnosis and Assessment of Cure Approaches for Acute Schistosomiasis in Pre-School Children
Source: Front Immunol. 2021 May 12;12:624736. doi: 10.3389/fimmu.2021.624736 (PMC8149760; doi:10.3389/fimmu.2021.624736)

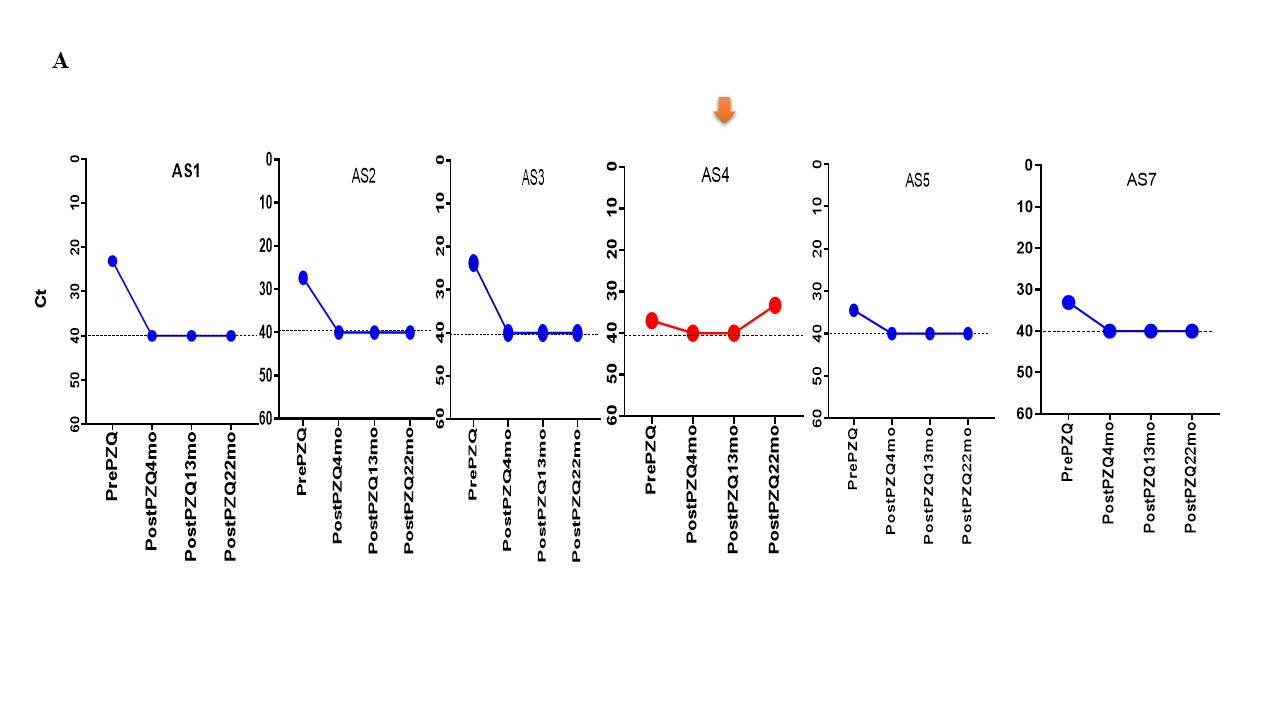

Supplement: Supplementary Figure 1 — Schematic representation of Schistosoma DNA, IgG and urinary antigen detection pre and post-treatment response during short and long-term follow up. Fecal samples from children treated with three sequential doses of PZQ (day 0, 30 and 45) were tested by real-time PCR (A). Serum (B) and urine (C) samples were analyzed by ELISA-IgG anti-SMMA and POC-CCA, respectively. Ct, Cycle Threshold (real-time PCR); Ct < 38, Reactive; Ct >38, Not Reactive; A.U., Arbitrary Units; ELISA IgG, Positive ≥ 1 AU. Negative < 1 AU; POC-CCA, 0, negative; 1, positive very weak (trace); 2, positive weak; 3, positive strong. Arrows indicate individuals with persistent reactivity post-drug use during long-term follow-up. In the 1st row, dashed lines represent the cut-off cycle threshold value. In the 2nd row, ELISA’s values > 1.0 were considered positive (dashed line). In the 3rd row, dashed lines correspond to results of the last urine sample (collected after 22 months of treatment) tested by POC-CCA test 2 (see Material and Methods). [file Image_1.jpeg]

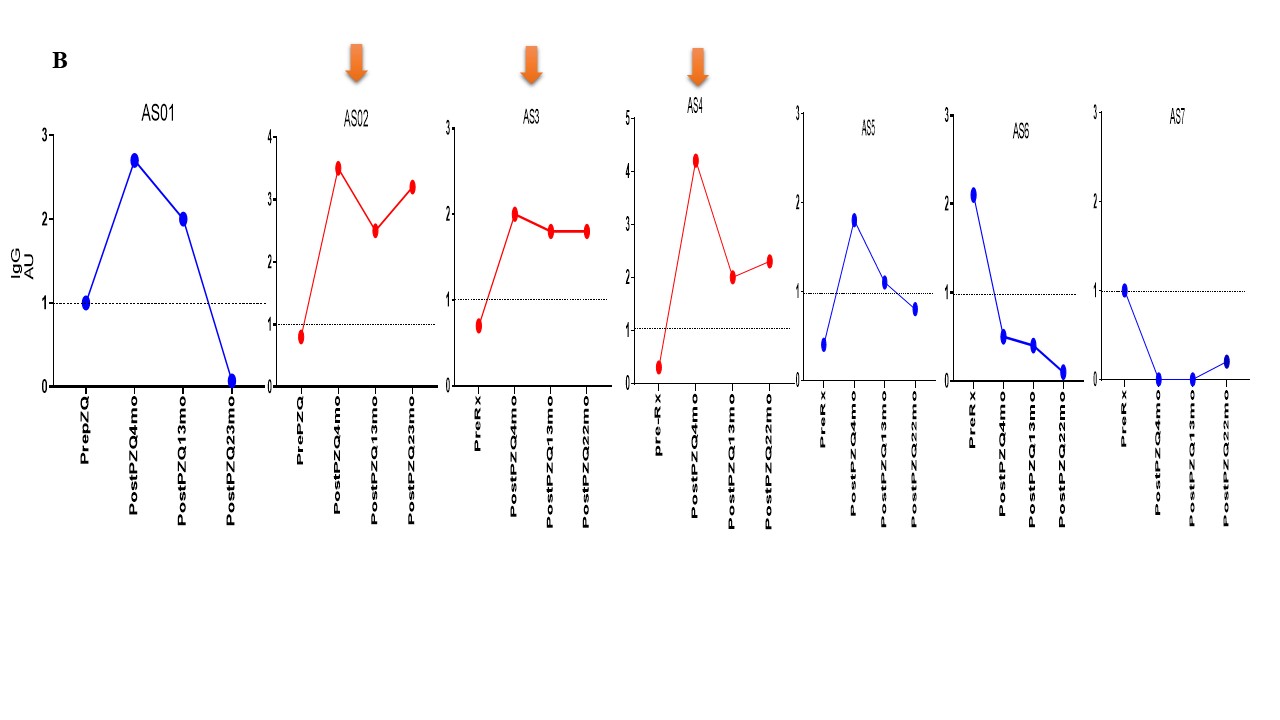

Supplement: Supplementary file 2 [file Image_2.jpeg]

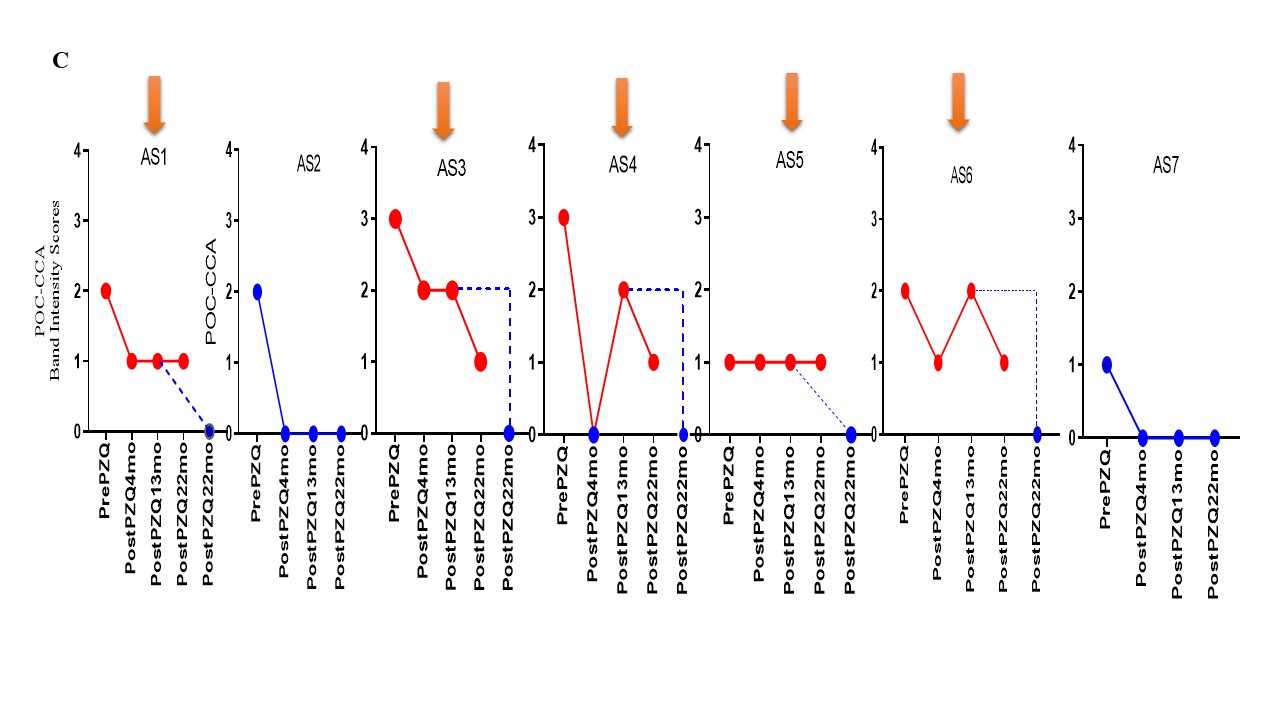

Supplement: Supplementary file 3 [file Image_3.jpeg]
